# Characteristics of men who have casual sex with men among Chinese university students: A cross-sectional study

**DOI:** 10.1371/journal.pone.0301817

**Published:** 2024-04-11

**Authors:** Weiyong Chen, Qiaoqin Ma, Xiaohong Pan, Lin Chen, Hui Wang, Xin Zhou, Tingting Jiang, Wanjun Chen

**Affiliations:** Department of HIV/STI Control and Prevention, Zhejiang Provincial Center for Disease Prevention and Control, Hangzhou, Zhejiang Province, China; Brown University, UNITED STATES

## Abstract

**Objectives:**

The characteristics of men who have sex with men (either exclusively or with both men and women; MSM) who engaged in casual sex among Chinese male university students have not been compared with the characteristics of men who have sex with only women (MSW). This information is important for tailoring targeted behavioral interventions to prevent human immunodeficiency virus (HIV)/sexually transmitted infection (STI) transmission in this subgroup of MSM.

**Methods:**

Data were derived from a large cross-sectional electronic questionnaire survey conducted at 13 universities in Zhejiang Province, China, in 2018. Bivariate analyses were used to compare demographic, HIV-related psychosocial, and behavioral characteristics between MSM and MSW students who engaged in casual sex during the previous year. Proportion differences between the two groups and their 95% confidence intervals were analyzed.

**Results:**

Among the 583 sexually active male students who engaged in casual sex during the previous year, 128 and 455 were MSM and MSW, respectively. Compared with MSW students, larger proportions of MSM students reported knowing that male-to-male sexual behavior was the main mode of HIV transmission among Chinese students (62.5% vs. 45.5%), consenting to commercial sex (67.2% vs. 53.4%), wanting to know the HIV serostatus of partners before casual sex (65.8% vs. 51.3%), feeling at risk of HIV infection (40.5% vs. 11.8%), high condom-decision scale scores (55.3% vs. 42.6%), engaging in sex with ≥ 5 casual sex partners (44.6% vs. 25.9%), searching for casual partners online (89.2% vs. 51.3%), consuming alcohol before casual sex (64.8% vs. 45.0%), engaging in sex with regular partners (83.1% vs. 67.0%), engaging in commercial sex (54.2% vs. 26.4%), and visiting a clinic for voluntary counselling and testing (VCT) (16.4% vs. 8.4%). However, compared with MSW students, smaller proportions of MSM students reported knowing that consistent condom use could prevent HIV transmission (80.5% vs. 95.2%) and that VCT should be actively sought after risky sexual behavior (78.9% vs. 93.8%), using condoms sometimes/often (26.4% vs. 44.3%), and consistently using condoms (28.9% vs. 40.1%) while engaging in sex with casual partners.

**Conclusions:**

MSM students who engaged in casual sex were at a greater risk of HIV/STI transmission, compared with MSW students. Comprehensive interventions to address the risks of unprotected male-to-male sex, searching for casual sex partners online, and non-use of HIV testing services are needed to reduce the burden of HIV/STI transmission among this subgroup of MSM.

## Introduction

The human immunodeficiency virus (HIV) epidemic continues to expand among men who have sex with men (MSM) in China. The national HIV prevalence among MSM in China was 8.0% in 2015, 200-fold greater than the 0.04% in the general Chinese population [1]. From 2001 to 2018, HIV prevalence increased among MSM in China [2]. The overall prevalence among MSM increased from 0.9% in 2003 to 8.0% in 2015 [3, 4]. This increasing trend of HIV prevalence among MSM highlights a unique risk population, which may influence the transmission of HIV in this country.

Regarding male students who have sex with men (a subgroup of MSM), a Chinese meta-analysis reported an HIV prevalence of 3.8% among high school and college MSM students [5]. Another meta-analysis showed that the prevalences of HIV and syphilis among Chinese high school and college MSM students were 4.4% (95% confidence interval [CI]: 3.0–6.4%) and 5.7% (95% CI: 4.8–6.7%), respectively [6]. Furthermore, HIV prevalence among MSM students across China is reportedly increasing; it was 3.0% in 2003–2006, 4.5% in 2007–2008, and 6.8% in 2009–2010 [6]. The number of HIV-infected students aged 15–24 years has increased annually over the past decade; more than 3,000 students in China have been infected during each of the past 2 years. Among them, the number of infected male students increased rapidly, while the number of infected female students remained stable (50–60 cases per year) [7]. HIV is mainly transmitted among students through unprotected male-to-male sexual intercourse, which constitutes approximately 80–90% of the infections [7–9]. In Beijing, this type of transmission led to > 85% of newly diagnosed HIV infections among students in 2016 [10].

Sex with regular, casual, or commercial partners; sex with multiple partners; and inconsistent condom use are common among MSM students in China, all of which place these students at risk of contracting HIV and other sexually transmitted infections (STIs) [11, 12]. Factors that increase the vulnerability of Chinese MSM students to HIV/STI transmission include discrimination against homosexuality in Chinese society, lack of sexual health education in schools, and lack of basic sexual health knowledge [13]. In addition, increased Internet use for social networking facilitates partner switching and casual sex [14–16]. University students are more vulnerable to HIV infections because they have greater freedom and personal space to socialize in the MSM community, while high school students are under parental supervision and experience heavy pressure to prepare for the National College Entrance Examination [17].

Numerous studies have assessed HIV-related knowledge, sexual behavior, HIV testing, substance use, and HIV infection status among MSM university students in China. Some studies have shown the higher risk of HIV infection among MSM university students when compared with non-MSM university students [12]. However, there is limited literature regarding MSM students who engage in casual sex (i.e., ever having sex with at least one casual partner). Not to mention characterizing MSM students by comparing the difference in HIV infection risk between MSM and non-MSM among university students who have casual sex. A Chinese study showed that 49.0% of young MSM had engaged in casual sex during the previous six months. Sex with multiple partners and inconsistent condom use during oral and anal sex were both more common among young MSM who had casual sex than among young MSM who did not. Casual sex was also associated with drug use and frequent Internet use [18]. HIV-infected MSM were more likely to find a male casual partner online or in gay venues than uninfected MSM [19]. Approximately 80% of HIV-positive MSM students reported that they had been infected through sexual intercourse with a male casual partner; all HIV-positive MSM who had casual sex reported that they found sexual partners online [20]. Therefore, an accurate understanding of the characteristics of MSM students who engage in casual sex is critical to prevent HIV transmission in this population. Using baseline data from a large survey conducted at 13 universities in Zhejiang Province, we compare the psychosocial characteristics and sex-related behaviors of MSM students with those of male students who have sex with only women (MSW) in an attempt to identify factors that are more or less likely to be associated with HIV transmission among MSM students.

## Materials and methods

### Study setting

In 2018, the Zhejiang Provincial Educational Bureau and the Zhejiang Provincial Commission for Health and Family Planning jointly initiated a pilot program for HIV prevention among approximately 176,000 students from 13 universities. The principal objectives for this program were to promote the construction of a leadership and coordination mechanism for HIV prevention and control in these universities, to promote healthy sexual behavior among students, to explore universal education and behavioral intervention models for all students that could raise awareness of HIV risk and self-protection, and to promote protective behaviors for HIV prevention [21]. These universities were distributed in all 11 municipalities of Zhejiang Province: 3 in Hangzhou (the provincial capital) and 1 in each of the other 10 municipalities. The universities were recommended by the local Centers for Disease Control and Prevention (CDC). In 2018, there were 107 universities in Zhejiang Province; the participation rate for this pilot program was 12.1% (13/107).

### Recruitment

The current study was a baseline survey for the pilot program conducted in October 2018 and November 2018. The survey was conducted using a stratified cluster sampling method. Three schools were first selected from each university via simple random sampling. Each selected school was naturally categorized into 4 strata, from Year 1 to Year 4; simple random sampling was then used to draw classes. The planned number of students to be recruited per year in each school was 200. In China, the number of students for each class was approximately 40–50. The number of classes surveyed per year within each school was based on the requirement of 200 students. The planned recruitment number was 2400 (200 per year × 4 years × 3 schools) in each university, for a total of 31,200 participants (2400 per university × 13 universities).

Electronic questionnaire surveys were conducted at all universities. The electronic questionnaire was generated using an online questionnaire editing software. A quick response (QR) code or web link for the electronic questionnaire was distributed via WeChat. The electronic questionnaire could also be accessed by scanning an offline QR code.

The investigators comprised counselling teachers who were responsible for these classes; all teachers were trained before the survey by a research team. The trained counselling teachers explained the purpose of the study, the survey methodology, and the privacy protection policy, and invited all students in the selected class to participate in the survey, but it is up to the student to decide whether or not to participate; they then distributed the questionnaire to each participant via WeChat and or by asking them to scan an offline QR code during a class meeting. Students were asked to complete the questionnaire online in a private space without any time limit.

### Participants

Sexually active male students who reported the types of their sexual partners during the previous year were included in this analysis. The types of sexual partners were categorized as regular(sex with boyfriend or girlfriend), casual(sex with ordinary acquaintances and with the people found on the internet, one-night stand), and commercial(sex involved in money or material transactions).

Students who reported engaging in casual sex, including ever having sex with only casual partners, casual and regular partners, regular and commercial partners, and causal, regular and commercial partners, were included in the analysis; they were divided into MSM and MSW groups. The MSM group comprised sexually active male students who engaged in sex with only men or both men and women. The MSW group comprised sexually active male students who engaged in sex with only women; this was used as a comparison group.

### Questionnaire development

Based on a review of research literature from China and the international community, as well as frequent discussion and modification, the questionnaire was developed by the research team. A pilot survey was conducted in a university class. The final questionnaire included participants’ demographic characteristics, HIV-related knowledge, exposure to various educational programs, sexual attitudes, sexual behaviors, and knowledge of/experience with HIV testing services.

### Ethical considerations

Participants were informed that the study was designed to develop strategies for the prevention of HIV and STI transmission among students. They were informed that the survey was anonymous, their privacy would be strictly protected, participation was voluntary, and that participants’ personal data would not be reported. The study was approved by the ethics committee of Zhejiang Provincial CDC (approval number: 2018–036). The research protocol was also approved by each participating university. Written informed consent was obtained from each participant and recorded by clicking the “agree” (in Chinese) button at the beginning of the electronic questionnaire. No participants were aged < 18 years. The study was performed in accordance with the relevant guidelines and regulations.

### Measures

Self-reports of sex with men among sexually active male students who engaged in casual sex during the previous year were analyzed based on demographic and psychosocial characteristics, sexual behaviors, condom-decision scale score, and factors associated with use of voluntary counselling and testing (VCT) services; these data were compared between the MSM and MSW groups.

The demographic variables included gender (male or female), age (years), residence (province and city), type of hometown (rural, urban, or suburban), and monthly living expenses (in yuan [RMB]).

Variables that reflected psychosocial characteristics included the following: male-to-male sexual behavior is the main method by which Chinese students contract HIV (correct, incorrect, or unknown), consistent condom use can prevent HIV transmission (correct, incorrect, or unknown), VCT should be actively sought after risky sexual behavior (i.e. multiple partners, commercial sex, casual sex, male-to-male sex, condomless sex)(correct, incorrect, or unknown), one-night stand acceptability (acceptable, unacceptable, or unknown), commercial sex acceptability (acceptable, unacceptable, or unknown), whether the participant wanted to know their partner’s HIV serostatus before casual sex (yes, no, or indifferent), and whether they perceived a risk of HIV infection (yes, no, or unknown). The condom-decision scale comprised the following 3 statements: “Are you able to discuss the use of condoms with your partner before sex?”; “Are you able to reject sex when your partner refuses to use a condom?”; and “Are you able to prepare a condom before sex?”. The possible responses to these 3 statements were “extremely able,” “very able,” “able,” “unable,” and “extremely unable,”; these were assigned scores of 3, 2, 1, 0, and −1, respectively. The combined score for this scale ranged from 0 to 9, where 9 reflects a high condom-decision level, 5–8 reflects a moderate condom-decision level, and 0–4 reflects a low condom-decision level. The Cronbach’s alpha coefficient for this scale was 0.771.

Variables related to HIV-related behavior during the previous year and their alternative answers included the following: the participant’s number of casual partners (specific number) category of casual partners (only students, only non-students, or both), online search for casual partners (yes or no), alcohol consumption before casual sex (yes or no), condom use with casual partners (never, sometimes, often, or always), sex with regular partner (yes or no), condom use with regular partner (never, sometimes, often, or always), category of regular partner (only students, only non-students, or both), engagement in commercial sex (yes or no); and, participants were also asked whether they had ever visited a VCT clinic (yes or no) during the life.

### Statistical analyses

Bivariate analysis was performed using the SPSS Statistics software (version 21.0; IBM Corp., Armonk, NY, USA). Proportion differences (PDs) for demographic, HIV-related psychosocial, and behavioral variables between the two groups, along with 95% confidence intervals (CIs), were computed using VassarStats software (an online statistical tool). The chi-squared test was used to compare differences between the MSM and MSW groups. P-values < 0.05 were considered statistically significant.

## Results

A total of 31,674 students participated in this baseline survey, including 14,320 men and 17,354 women. Of the 2,734 sexually active men, 2,665 sexually active men who reported the types of their sexual partners during the previous year were included in this analysis.

Among the 2,665 sexually active male students, 219 were MSM and 2,446 were MSW. Casual sex was reported by 583 of these 2,665 students, including 128 MSM and 455 MSW. Thus, the proportions of MSM and MSW who engaged in casual sex in this study were 58.4% (128/219) and 18.6% (455/2446), respectively. The 128 MSM who engaged in casual sex constituted 22.0% (128/583) of the male sexually active students who had engaged in casual sex during the previous year.

### Demographic characteristics of participants who engaged in casual sex

Table 1 shows the participants’ demographic characteristics. Most of the participants were aged 20–21 years and were year 2 or 3 students. Moreover, 43.8% and 56.5% of the participants were from rural areas in the MSM and MSW groups, respectively; most of these participants were from Zhejiang Province. Approximately two-thirds of the participants had monthly living expenses > 1000 Yuan. There were no significant differences between the two groups in terms of residence and monthly living expenses. Compared with MSW students, MSM students were significantly more likely to be 20–21 years old (PD = 10.3%, 95% CI = 0.5–19.5%, P = 0.045) and to be from a suburban or urban area (PD = 12.7%, 95% CI = 2.9–22.2%, P = 0.012), whereas they were significantly less likely to be year 1 students (PD = 9.1%, 95% CI = 1.3–9.2%, P = 0.025).

**Table 1:** Demographic characteristics of sexually active male students who engaged in casual sex.

	MSM (N = 128)	MSW (N = 455)
	N^a^, %	N^a^, %
Age		
≤ 19 years	35 (27.3)	156 (34.3)
20–21 years	77 (60.2)	227 (49.9)
≥ 22 years	16 (12.5)	72 (15.8)
Year		
One	17 (13.3)	102 (22.4)
Two	41 (30.0)	155 (34.1)
Three	50 (39.1)	152 (33.4)
Four	20 (15.6)	46 (10.1)
Residence		
Zhejiang Province	100 (78.7)	327 (71.9)
Other province	27 (21.3)	128 (28.1)
Hometown		
Rural	56 (43.8)	257 (56.5)
Suburban/urban	72 (56.3)	198 (43.5)
Living expenses		
≤ 1000 Yuan	47 (36.7)	135 (29.7)
1001–1500 Yuan	31 (24.2)	135 (29.7)
≥ 1501 Yuan	50 (39.1)	184 (40.6)

^a^Numbers may not sum to 128 for MSM and 455 for non-MSM because of missing data.

### Psychosocial characteristics of MSM who engaged in casual sex

Compared with MSW students, MSM students were significantly more likely to know that unprotected male-to-male sexual behavior is the main method by which Chinese students contract HIV (62.5% vs. 45.5%; P = 0.001), to consent to commercial sex (67.2% vs. 53.4%; P = 0.006), to want to know the HIV serostatus of their partner before casual sex (65.8% vs. 51.3%; P = 0.005), to feel at risk of HIV infection (40.5% vs. 11.8%; P = 0.000), and to have a high condom-decision scale score (55.3% vs. 42.6%; P = 0.014). They were also significantly less likely to think that consistent condom use could prevent HIV transmission (80.5% vs. 95.2%; P = 0.000), to know that VCT should be actively sought after risky sexual behavior (78.9% vs. 93.8%; P = 0.000), and to have a moderate condom-decision scale score (22.8% vs.34.4%; P = 0.016) (Table 2). There were no significant differences between the two groups in terms of one-night stand acceptability.

**Table 2:** Psychosocial characteristics of MSM and MSW among male sexually active students who engaged in casual sex.

	MSM (N = 128)	MSW (N = 455)	PD (95% CI)^b^	P*-*value^c^
	N^a^, %	N^a^, %		
Male-to-male sexual behavior is the main method by which Chinese students contract HIV				
Incorrect/unknown	48 (37.5)	248 (54.5)		
Correct	80 (62.5)	207 (45.5)	17.0 (6.8–26.5)	0.001
Consistent condom use could prevent HIV transmission				
Incorrect/unknown	25 (19.5)	22 (4.8)		
Correct	103 (80.5)	433 (95.2)	14.7 (8.0–23.0)	0.000
VCT should be actively sought after risky sexual behavior				
Incorrect/unknown	27 (21.1)	28 (6.2)		
Correct	101 (78.9)	427 (93.8)	14.9 (7.9–23.5)	0.000
One-night stand				
Unacceptable/unknown	29 (22.7)	100 (22.0)		
Acceptable	99 (77.3)	355 (78.0)	0.7 (−7.2–9.9)	0.870
Commercial sex				
Unacceptable/unknown	42 (32.8)	212 (46.6)		
Acceptable	86 (67.2)	243 (53.4)	13.8 (3.7–23.0)	0.006
Want to know the HIV serostatus of partner before casual sex				
No/indifferent	41 (34.2)	205 (48.7)		
Yes	79 (65.8)	216 (51.3)	14.5 (4.1–24.1)	0.005
Risk perception for HIV infection				
No/unknown	72 (59.5)	373 (88.2)		
Yes	49 (40.5)	50 (11.8)	28.7 (19.3–38.0)	0.000
Condom-decision scale score				
0–4	27 (22.0)	99 (23.0)	1.1 (−8.3–9.1)	0.810
5–8	28 (22.8)	148 (34.4)	11.7 (2.0–20.0)	0.016
9	68 (55.3)	183 (42.6)	12.7 (2.3–22.3)	0.014

^a^Numbers may not sum to 128 for MSM and 455 for non-MSM because of missing data.

^b^PD, Proportion difference; CI, confidence interval.

^c^P-values were calculated using the chi-squared test.

### HIV-related behavioral characteristics of MSM who engaged in casual sex

Compared with MSW students, MSM students were significantly more likely to have engaged in sex with ≥ 5 casual sex partners during the previous year (44.6% vs. 25.9%; P = 0.000), to search for casual partners online (89.2% vs. 51.3%; P = 0.000), to consume alcohol before casual sex (64.8% vs. 45.0%; P = 0.000), to never use a condom (67.0% vs. 16.3%; P = 0.000), to engage in sex with regular partners (83.1% vs. 67.0%; P = 0.001), to engage in commercial sex (54.2% vs. 26.4%; P = 0.000), and to visit a VCT clinic (16.4% vs. 8.4%; P = 0.009), whereas they were significantly less likely to sometimes/often use condoms (26.4% vs. 44.3%; P = 0.000) and to consistently use condoms (28.9% vs. 40.1%; P = 0.026) while engaging in casual sex (Table 3). Among students who also engaged in sex with regular partners, MSM students were significantly more likely to never use a condom (67.0% vs.16.3%; P = 0.000) and to engage in sex with non-students (14.6% vs. 5.8%; P = 0.001), whereas they were significantly less likely to sometimes/often use condoms (14.6% vs. 47.1%; P = 0.000), consistently use condoms (18.4% vs. 36.6%; P = 0.000), and engage in sex with only students (67.0% vs. 79.1%; P = 0.011), compared with MSW students.

**Table 3:** HIV-related behavioral characteristics of MSM and MSW among male sexually active students who engaged in casual sex.

	MSM (N = 128)	MSW (N = 455)	PD (95% CI)^b^	P*-*value^c^
	N^a^, %	N^a^, %		
Number of casual partners during the previous year				
1	32 (31.7)	150 (39.7)	8.0 (−3.3–18.1)	0.166
2–4	24 (23.8)	130 (34.4)	10.6 (−0.2–19.8)	0.055
≥ 5	45 (44.6)	98 (25.9)	18.6 (7.7–29.7)	0.000
Categories of casual partners during the previous year				
Students	78 (63.9)	280 (66.2)	2.3 (−7.4–12.5)	0.746
Non-students	21 (17.2)	57 (13.5)	3.7 (−3.3–12.4)	0.306
Both	23 (18.9)	86 (20.3)	1.5 (−7.6–9.1)	0.798
Searched online for casual partners during the previous year				
No	13 (10.8)	206 (48.7)		
Yes	107 (89.2)	217 (51.3)	37.9 (29.1–44.6)	0.000
Used alcohol before casual sex during the previous year				
No	43 (35.2)	233 (55.0)		
Yes	79 (64.8)	191 (45.0)	19.7 (9.3–29.3)	0.000
Used condom with casual partner during the previous year				
Never	54 (44.6)	66 (15.6)	29.1 (19.3–38.9)	0.000
Sometimes/often	32 (26.4)	188 (44.3)	17.9 (7.8–26.8)	0.000
Always	35 (28.9)	170 (40.1)	11.2 (1.0–20.3)	0.026
Engaged in sex with a regular partner during the previous year				
No	21 (16.9)	146 (33.0)		
Yes	103 (83.1)	296 (67.0)	16.1 (7.0–23.6)	0.001
Used condom with regular partner during the previous year (n = 399)				
Never	69 (67.0)	48 (16.2)	50.7 (39.6–60.3)	0.000
Sometimes/often	15 (14.6)	139 (47.0)	32.6 (22.2–40.9)	0.000
Always	19 (18.4)	108 (36.5)	18.2 (7.5–27.0)	0.000
Category of regular partner during the previous year (n = 399)				
Students	69 (67.0)	234 (79.1)	12.6 (2.4–23.6)	0.011
Non-students	15 (14.6)	17 (5.7)	8.8 (1.9–17.7)	0.007
Both	19 (18.4)	43 (14.5)	3.8 (−4.4–13.7)	0.430
Ever engaged in commercial sex				
No	55 (45.8)	312 (73.6)		0.000
Yes	65 (54.2)	112 (26.4)	27.8 (17.4–37.7)	0.000
Ever visited a VCT clinic				
No	107 (83.6)	417 (91.6)		
Yes	21 (16.4)	38 (8.4)	8.1 (1.6–16.2)	0.009

^a^Numbers may not sum to 128 for MSM and 455 for non-MSM because of missing data.

^b^PD, Proportion difference; CI, confidence interval.

^c^P-values were calculated using the chi-squared test.

## Discussion

Our study examines a range of characteristics of MSM students who engage in casual sex, using MSW as a comparison group. To our knowledge, this is the first of its kind in China. Sex with casual partners is itself a risky behaviour for contracting HIV/STIs if it is not well protected. Our study found that MSW students who engaged in casual sex had a risk profile that included accepting one-night stands and commercial sex, low risk awareness of HIV infection, multiple sexual partnerships, searching for casual partners online, using alcohol before casual sex, low condom use, ever having commercial sex, and having sex with a regular partner, all of which were not low in proportion. However, our MSM students showed a much higher level of vulnerability to the transmission of HIV/STIs in these respects when comparing to MSW students.

Our study revealed that 58.4% of MSM students engaged in casual sex during the previous year; similarly, a respondent-driven sampling study of MSM in Zhejiang Province showed that 61.3% of MSM had engaged in casual sex with another man during the previous half year [22]. However, the proportion in our study was significantly higher than the proportion in another Chinese study of MSM in 2018–2019, which found that 26.2% of MSM students had engaged in casual sex during the previous half year [15]. The prevalence in our study was also significantly higher than the 28.6% we observed in a previous study concerning sexual behavior among Chinese MSM students during the previous year [12]. The high prevalence of casual sex among MSM students is concerning because it carries a high risk of transmitting HIV and other STIs.

We found that 61% of MSM students consumed alcohol before engaging in casual sex; this was significantly greater than the proportion among MSW students. Alcohol use reportedly increases the likelihood of risky sexual behavior. An epidemiological survey and meta-analysis of alcohol use among Chinese MSM reported that the pooled prevalence of alcohol use was 32%, while the pooled prevalence of drinking more than once a week and drinking before sex with male partners was 23%. MSM who consumed alcohol more than once weekly were more likely to use drugs, have sex with women, have unprotected insertive or receptive anal sex with men, have more than 10 lifetime male sex partners, predominantly practice insertive anal sex, and trade sex for money, compared with MSM who consumed alcohol less than once weekly [23]. Other studies have reported that alcohol consumption among Chinese MSM was associated with higher odds of condomless insertive or receptive anal sex and multiple male partners [24, 25]; there was a dose-response relationship between the alcohol use and engagement in condomless anal sex [24]. Our study demonstrated that the proportions of alcohol use before casual sex and various related risk factors for HIV infection were significantly greater among this subgroup of MSM. This finding implies a need to reduce the harm from alcohol use, specifically in terms of education about the risks related to alcohol use among MSM students.

The Internet provides a convenient platform for MSM to find sexual partners; it is now a major venue used by Chinese MSM to search for sexual partners [2]. Casual partners of all MSM students [16],and particularly MSM who engaged in casual sex [20],are mainly found online. In our study, 89.2% of MSM students searched for casual sexual partners online; these students have higher overall probabilities of unprotected anal sex, sex with multiple partners, and contracting STIs [5, 18, 26]. A Chinese survey indicated that all HIV-positive MSM students who became infected through casual sex with another man had sought sexual partners online [20]. Most MSM students in our study indicated that one-night stands were acceptable. These findings highlight the specific HIV risk-related behaviors of MSM students in terms of seeking casual sex partners online; they indicate a need for social media interventions to mitigate the enhancing effects of such risk-related behaviors on HIV transmission in MSM students.

Although MSM students demonstrated high levels of knowledge regarding male-to-male sexual intercourse as the main method by which Chinese students contract HIV, and regarding the potential for consistent condom use to prevent HIV transmission, they reported surprisingly low rates of consistent condom use with casual and regular partners; thus, their high levels of safe sex knowledge were not directly associated with safe sexual behaviors. Only 55.3% of MSM students had a high condom-decision scale score. This finding indicates that knowledge and education are insufficient for behavioral change. MSM students exposed to HIV interventions were less likely to be infected with HIV, compared with MSM not exposed to HIV interventions [27]. A systematic review and meta-synthesis of qualitative studies using the social-ecological model identified barriers to condom use among MSM, including physical discomfort, lack of knowledge about HIV/STIs and substance use, condom stigma (i.e. using condoms as a symbol of mistrust or HIV/STI prevention, as a violation of traditional notions of sex, or as an embarrassing topic), situational unavailability, unaffordability of condoms, and power imbalance in the sexual relationship [28]. The main reason for the low rate of consistent condom use among the MSM population was condom-related stigma [29]. Understanding the various barriers to condom use among MSM students, particularly condom-related stigma, and developing multifactorial interventions based on the identified barriers, is critical for an effective condom promotion program.

HIV risk perception was significantly greater among MSM students than among MSW students. Additionally, MSM students were more likely to want to know the serostatus of their casual partner; this is presumably because MSM students are more aware of their own HIV infection risk than MSW due to their risky sexual behaviors. A previous study suggested that MSM who engage in unprotected anal sex are more likely to report moderate/high perceived risk of HIV [30]; additionally, MSM with a high perceived risk of HIV were more likely to be HIV-positive [22]. However, HIV risk perceptions were generally low among MSM students in our study who engaged in casual sex (40.5%). The Health Belief Model and others [31] consider risk perception to be an important predictor of risky behaviour. MSM perceive themselves to be at low risk of HIV infection, when in fact they are at high risk [32, 33]. In addition, low risk perceptions among young people lead to reduced condom use and HIV testing. This increases their risk of HIV infection [34]. Risk awareness education should be an essential component of HIV prevention programs among Chinese students, and the vulnerability of MSM to HIV transmission at the individual and community levels should be emphasized. Among MSM who engaged in casual sex, we found that 83% engaged in sex with a regular partner during the previous year. MSM in the United States are mainly infected with HIV from their regular partners [35]. In China, although HIV infections among MSM are mainly associated with sex with casual partners [19, 20], the proportion of new HIV infections attributed to sex with regular partners has increased [36]. A cohort study demonstrated that unprotected anal sex with regular partners was a predictor of HIV seroconversion among Chinese MSM [37]. The rate of consistent condom use was significantly lower with regular partners than with casual partners among MSM in this study (18.4% vs. 28.9%), similar to the findings in another study of MSM in Zhejiang Province [22]. MSM students may not be aware of the infection risk from their male regular partners. Thus, MSM students who engage in sex with casual partners are also at risk of HIV infection from their regular partners; they need education concerning potential HIV risks related to their regular partners.

In addition to the high proportion of MSM with regular partners, nearly half of MSM students also engaged in commercial sex during the previous year; the proportion was significantly greater among MSM students than among MSW students. Another issue of concern is sex with multiple partners. MSM students were more likely to have > 5 casual partners during the previous year, compared with MSW students. Engagement in sex with multiple partners, while using a low level of protection, may be associated with HIV infections in Chinese MSM students [27]. Among MSM students in our study, casual partners included both students and non-students; their regular partners also included a greater proportion of non-students. The high proportion of multiple, commercial and regular partners, and inconsistent condom use reported in this study, together with the high prevalence of HIV and syphilis among Chinese MSM students [6, 38], suggest that a high-risk sexual network for HIV/STI transmission has been established among students. HIV prevalence and incidence were much higher among MSM students’ social contacts than among MSM students themselves, and HIV-positive MSM are more likely to be hyperconnected within the student social network than HIV-negative MSM [38]. This sexual network, linking students and non-students, could lead to a more severe HIV epidemic, as in the case of MSM in the community [22, 38], if it is not effectively addressed as soon as possible. Among the MSM students in our study who engaged in casual sex, 33.6% also had sex with women. Studies in China have shown that men who engage in bisexual behaviour are more likely to engage in unprotected anal sex, have male commercial partners, use alcohol and drugs, are less likely to access HIV prevention and testing services, and have significantly higher HIV prevalence [39–41] than men who have sex only with men. Further studies are needed to understand the HIV-related risks of male students who engage in bisexual behaviour and their unmet needs for HIV prevention. Additionally, only 16% of MSM students had been exposed to VCT, and the rate of visits to VCT clinics remained low, although a greater proportion of MSM students visited VCT clinics compared with MSW students. In addition, the proportion of students who thought that VCT should be actively sought after a risky sexual behavior was significantly lower among MSM students than among MSW students. In China, 35–40% of HIV-positive MSM students are identified in VCT clinics [8, 9]. Exposure to HIV prevention interventions is an independent factor that influences the use of HIV testing services by MSM students [42] and MSM overall [43] in China. Increased knowledge of HIV and VCT clinics could promote the use of VCT services, while a lack of risk perception and a fear of HIV infection could discourage their use [44]. VCT is widely available—VCT facilities are present in local CDCs and hospitals in every county within Zhejiang Province. VCT services are confidential, free of charge, and performed by professionals [45]. Future HIV testing promotion programs implemented in Chinese universities should investigate the reasons for low VCT utilization among MSM students and aim to increase awareness of HIV risk, the benefits of HIV testing, and the characteristics of VCT.

The results of this study should be interpreted in light of several limitations. First, this was a cross-sectional survey; thus, causal relationships cannot be inferred. Second, all measures of sexual behaviors were based on self-report data from an electronic questionnaire, which may have led to reporting bias; however, efforts were made to reduce such bias by ensuring anonymity and privacy. Our sample comprised MSM and MSW students who engaged in sex with casual partners; therefore, students who felt uncomfortable disclosing such sexual behaviors may have been missed. Third, sexual health behaviour among MSM is strongly influenced by factors at the individual level, such as psychology, knowledge, attitudes, behaviour, factors at the interpersonal level, such as social networks, social support, and factors at the environmental/structural level, such as community, public policy, inter-organisational relationships, culture [46, 47]. Our study focuses mainly on describing individual-level risk factors; comprehensive research characterising multiple levels of HIV risk is needed in the future to inform the combination of HIV prevention packages. Finally, we did not perform a multivariate analysis to explore associated factors because of the limited sample size in this analysis.

## Conclusions

Despite some limitations, the findings in this study advance the understanding of the characteristics of MSM students who engage in casual sex. They were significantly more likely to have more casual partners, to search for sexual partners online, to engage in sex with regular and commercial partners, and to consume alcohol before engaging in casual sex, compared with MSW students; moreover, MSM students were less likely to use condoms, compared with MSW students. These findings indicate the presence of a complex sexual network that contributes to HIV/STI transmission among MSM students. Comprehensive interventions that address these risks and their associated factors are needed to reduce the burden of HIV/STI transmission in this uniquely vulnerable subgroup of MSM. The positive findings of this study, such as a higher proportion of MSM than MSW students wanting to know their partner’s HIV serostatus before having casual sex, a higher perceived risk of HIV infection, a higher condom decision score, and a higher proportion having ever visited a VCT clinic, and their backgrounds, should also be taken into account when promoting sexual health behaviours in such an intervention programme. In addition, although MSW students are used as the comparison group, an intervention targeting them should not be ignored, as MSM students in this study do not have a low level of vulnerability to HIV/STI infection.
